# Human Endogenous Retroviruses and Type 1 Diabetes

**DOI:** 10.1007/s11892-019-1256-9

**Published:** 2019-11-21

**Authors:** Sandrine Levet, B. Charvet, A. Bertin, A. Deschaumes, H. Perron, D. Hober

**Affiliations:** 1 GeNeuro Innovation, 60 avenue Rockefeller, 69008 Lyon, France; 2 Faculté de Médecine, CHU Lille, Laboratoire de Virologie EA3610, Université Lille, F-59000 Lille, France; 3 Laboratoire des déficits immunitaires, University of Lyon, Lyon, France; 4 Plan-les-Ouates, GeNeuro SA, Geneva, Switzerland

**Keywords:** Endogenous retrovirus, Type 1 diabetes, HERV, Enterovirus, Coxsackievirus B4

## Abstract

**Purpose of the Review:**

The aim of this review is to discuss recent data pointing at an involvement of human endogenous retroviruses (HERVs) in type 1 diabetes (T1D) onset and progression.

**Recent Findings:**

The envelope protein of HERV-W family, named HERV-W-Env, was detected in pancreata from T1D patients and was shown to display pro-inflammatory properties and direct toxicity toward pancreatic beta cells.

**Summary:**

The etiopathogenesis of T1D remains elusive, even if conventional environmental viral infections have been recurrently involved. Nonetheless, a new category of pathogens may provide the missing link between genetic susceptibility and environmental factors long thought to contribute to T1D onset. A number of studies have now shown that HERV sequences, which are normally inactivated or repressed in the human genome, could be activated by environmental viruses. Thus, if similarly activated by viruses associated with T1D, disregarded HERV genes may underlie T1D genetic susceptibility. Moreover, once expressed, HERV elements may display broad pathogenic properties, which identify them as potential new therapeutic targets.

## Introduction

The etiopathogenesis of some diseases has remained elusive, particularly for those involving a complex interplay between environmental and genetic factors, such as autoimmune diseases. Past decades have witnessed the development of a new comprehensive approach for these multifactorial diseases, which involves the study of a new kind of pathogenic elements: human endogenous retroviruses (HERVs). In the current state of knowledge, HERV may fill the gap between environmental factors, genetic factors, and pathogenic mechanisms of diseases such as multiple sclerosis (MS) or amyotrophic lateral sclerosis (ALS), only quoting the most studied ones in this HERV paradigm. This review will address HERV biology, introduce their involvement in MS and ALS, and will then focus on the evidence suggesting a role for HERV in type 1 diabetes (T1D).

## A Disregarded Moiety of Human Genome May Enlighten the Etiopathogenesis of Some Autoimmune and Neurogenerative Diseases

### Mobile Genetic Elements and HERV

Nearly half of the human genome is composed of remnants from mobile genetic elements, which were considered as “junk DNA” at the dawn of genome sequencing. These elements are vestiges of ancient viral infections that occurred million years ago [1••]. Mobile genetic elements encompass transposons and retrotransposons, based on the intermediate used to integrate into the genome, DNA or RNA respectively [2, 3]. HERVs belong to the retrotransposon group and display LTR (long terminal repeats) at their extremities, which are regions involved in chromosomal retro-integration and in regulation of transcription [1, 4].

**Fig. 1:**
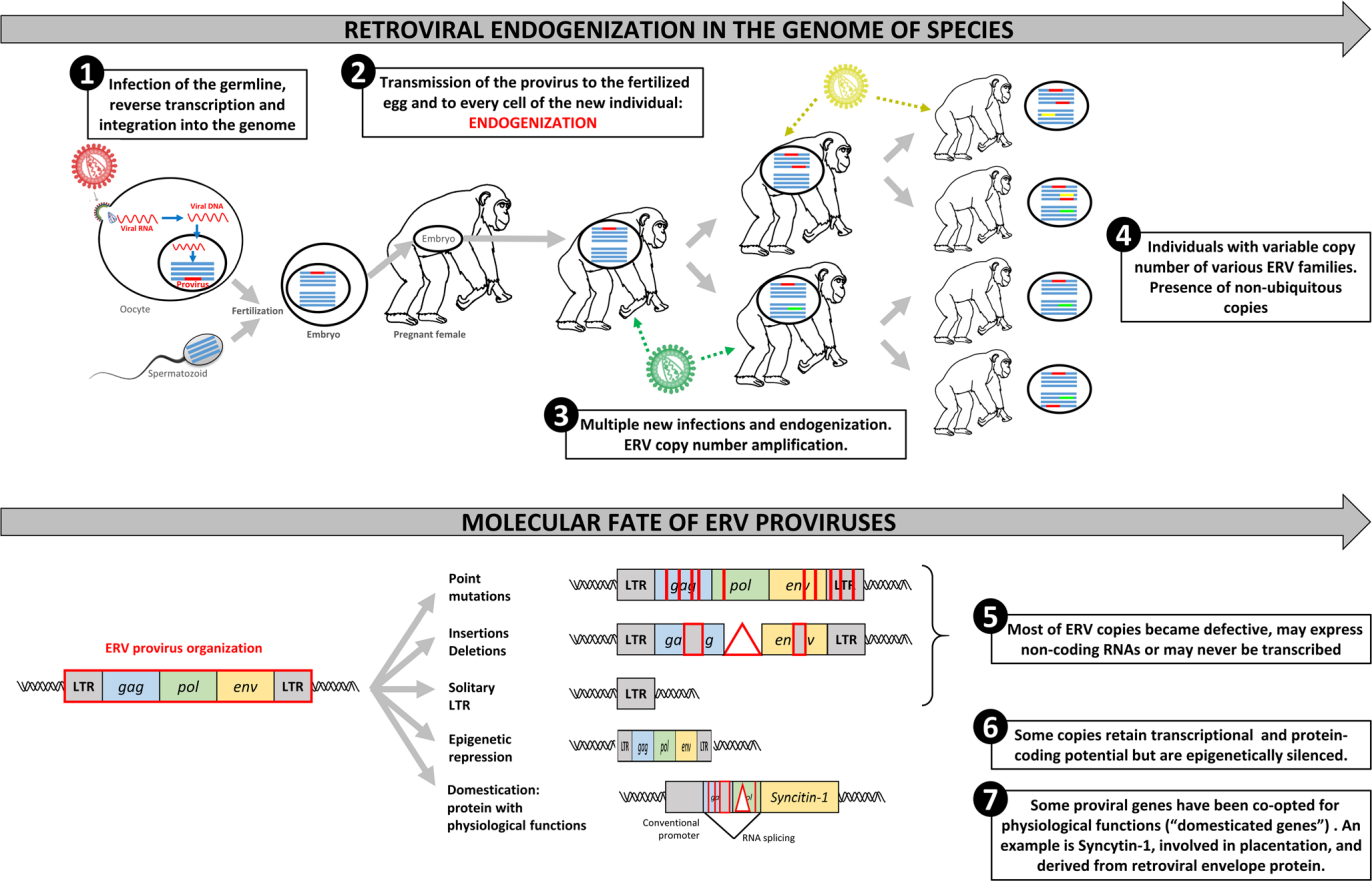
Endogenization of retroviruses during evolution. The upper panel depicts the successive steps leading to the stable insertion of environmental viral RNA into the genome of species. ❶ This process, called endogenization, starts from the infection of germ cells leading to the integration of the provirus into a chromosome. ❷ Fertilization with one of these infected cells will result in an individual harboring the provirus in each of its cells, both germinal and somatic. The provirus will then be transmitted to the offspring of this individual. ❸ Other environmental retroviruses can simultaneously undergo a comparable endogenization process, ❹ eventually leading to multiple and variable copy number in the final population. The lower panel illustrates some of the evolution options of a provirus once integrated into chromosomal DNA. ❺ Most of ERV copies will become defective, due either to mutations, deletions or insertions. ❻ For copies retaining protein coding potential, processes of epigenetic silencing, such as methylation, normally suppress transcriptional activity. ❼ Alongside the neutralization of the provirus described above, the evolution sometimes takes advantage of a viral function to enrich the physiological functions of the host. A well-known example is Syncytin-1 protein, which derives from an HERV-W envelope protein harboring fusogenic activity. Syncytin-1 is expressed during placentation and is required for trophoblast cells fusion into a multinucleated syncytiotrophoblast layer, a critical step of placental development

HERVs were generated by a process called “endogenization.” This happened when environmental retroviruses infected the germinal cells of hosts during evolution, leading to their transmission into the genome of the progeny and to next generations (Fig. 1). Owing to molecular properties of retroviral ancestors, HERVs are subjected to intra-genomic dissemination through retrotransposition and recombination, thus creating multiple copies of HERV genes [5]. Moreover, multiple new infections over very long periods of species evolution caused re-entries in the genomes of certain individuals, thus generating genomic diversity with newly integrated copies [6]. As a consequence, HERVs account for approximately 8% of the human genome, although most HERV copies are inactivated by mutations, deletions, or silenced by epigenetic modifications (Fig. 1) [1••].

With nearly two decades hindsight, it appeared that HERV copies are not mere “junk DNA.” Some of them have been domesticated and now belong to the pool of physiological genes, such as Syncytin-1 involved in placentation, while others have retained pathological properties when abnormally activated (Fig. 1).

### Activation of HERV

The prerequisite for an effective transcription is that HERV sequences must retain a functional LTR, not have major deletions and/or nucleotide substitutions, and must be epigenetically de-repressed. If these conditions are met, both micro- and macro-environmental triggers may activate HERVs.

#### Epigenetic Control

**Fig. 2:**
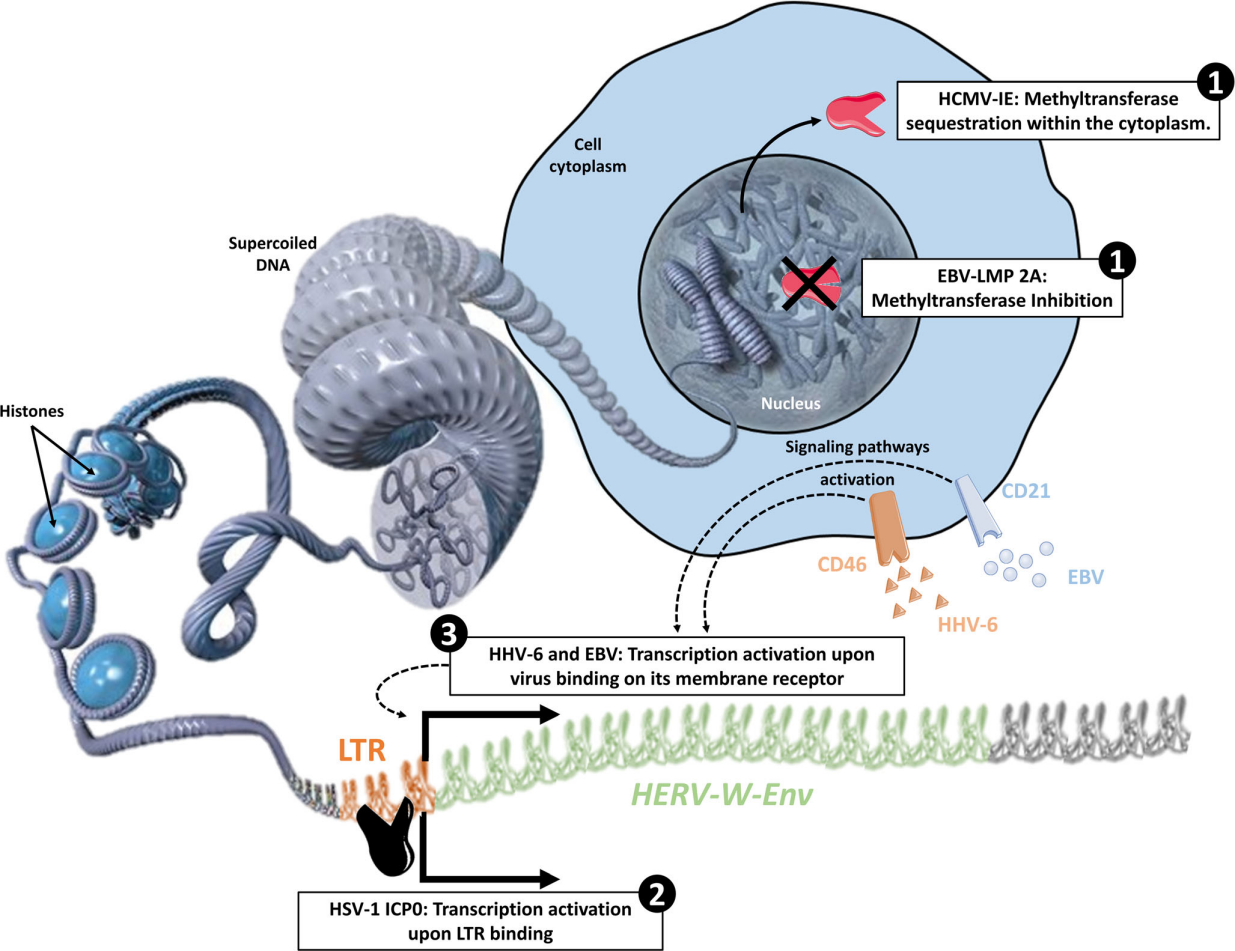
Dysregulation of epigenetic control and HERV transactivation by Herpesviridae. Mechanisms of transactivation by herpesviruses are exemplified with EBV (Epstein Barr virus), HSV-1 (herpes simplex type 1), HHV-6 (human herpesvirus 6), and HCMV (human cytomegalovirus). ❶ HERV copies are normally not accessible to transcription thanks to DNA methylation and to other mechanisms of epigenetic control. Herpesviruses can impair the methylation process, either by inhibiting methytransferases as shown for LMP-2A (latent membrane protein 2A) from EBV, or by sequestrating methyltransferases within the cytoplasm as shown for IE (immediate early) protein from HCMV. ❷ ICP0 (infected cells polypeptide 0) viral protein from HSV-1 were described to activate HERV-W LTR, thereby activating initiation of transcription. ❸ Upon binding of EBV and HHV-6 on their receptors, CD21 and CD46 respectively, signaling pathways activation leads to the transcription of HERV-W

HERV activation is linked to the chromatin state where the provirus is located (Fig. 2) [1••]. DNA methylation and histone modifications are key factors in the epigenetic control of the transcription of human genes as well as of HERV elements. EBV (Epstein Barr virus) and CMV (cytomegalovirus) have been shown to demethylate HERV sequences, thereby unlocking their epigenetic silencing [7, 8] and further allowing activation of normally “non-responding” copies when triggered by later occurrence of transactivating events.

#### Environmental Viruses

Environmental viruses have been shown to directly transactivate HERV elements in several studies (Fig. 2). HERV genes and/or reverse transcriptase (RT) activity are activated in various human cell types by *Herpesviridae*, including HSV-1 (herpes simplex virus type-1) [9, 10], VZV (varicella-zoster virus) [9], CMV [11], HHV-6 (human herpes virus type 6) [12], and EBV [13, 14]. EBVgp350 protein activated HERV-W in B cells and monocytes, but not in T cells or NK cells [14]. Thus, many viruses that have not previously been indicated as causative agents of autoimmune diseases were shown to trigger activation of HERVs, which in return may activate well-known pathogenic cascades in diseases such as MS, T1D, and ALS.

#### Inflammatory Stimuli

Inflammatory stimuli may activate epigenetically dysregulated HERV. Transcription of already activated HERV-W sequences may be upregulated by pro-inflammatory cytokines such as IL-6 (interleukin 6) and TNF-α (tumor necrosis factor alpha) [15], but are inhibited by IFN-β (interferon beta) [16] .

#### Drugs and Toxins

Antipsychotic drugs have been associated with activation of HERV transcription. Valproic acid, for example, may activate HERV-W transcription in human glioblastoma cell line SK-N-SH [17] and a pesticide named dieldrin may also activate HERV-driven enhancers in human T cells [18].

As described above, environmental factors have the ability to activate HERV copies otherwise hidden and silenced in the human genome. The consequence of this process can be deleterious and may result in diseases as exemplified with MS and ALS, in the forthcoming section.

### Examples of Best-Studied Associations Between HERV and Human Diseases

#### HERV-W and Multiple Sclerosis

MS provides an example of an autoimmune and neurodegenerative disease whose etiopathogenesis has recently been made more comprehensive by the discovery of the involvement of HERV. MS is an inflammatory disease of the central nervous system, causing myelin destruction and oligodendrocytes demise, followed by axonal disruption, neurodegeneration, and increasing disability. The etiology of MS lies somewhere at crossroads between genetic predisposition [19] and environmental factors, such as infectious mononucleosis and smoking [20].

Several HERV families have been linked to MS, including HERV-K, HERV-H, and HERV-W. However, a recent meta-analysis concluded that the most compelling association is with HERV-W [21]. HERV-W *env* and *pol* RNA have been detected in serum and PBMC (peripheral blood mononuclear cell) of MS patients [22, 23], and the presence in the CSF (cerebrospinal fluid) was associated with a poor prognosis of the disease [24]. HERV-W protein expression in active lesions has been detected in macrophages, astrocytes, and in endothelial cells of neighboring blood vessels [25], but was predominantly observed in microglial cells, the cell type driving the process leading to axonal demyelination, lesion expansion, and neurodegeneration [26••].

HERV-W-Env pathogenic functions have also been implicated both in inflammatory processes and in direct cytopathic effects affecting non-immune cells [26••]. HERV-W-Env induced the secretion of pro-inflammatory cytokines in vitro by human immune cells, a process requiring TLR4 (Toll-like receptor 4) activation [27]. In vivo, HERV-W-Env promoted anti-myelin autoimmunity leading to experimental autoimmune encephalomyelitis [28••] and elevated pro-inflammatory cytokine production upon systemic injection in mice [27]. HERV-W-Env was also found to mediate TLR4-dependent effects in non-immune cells such as oligodendrocytes precursor cells (OPC), which are prevented from differentiating upon HERV-W-Env exposure, thereby precluding their remyelinating functions [29]. Thus, converging data have identified the pathogenic protein HERV-W-Env as a therapeutic target in MS, and have prompted the clinical development of temelimab (formerly GNbAC1), a humanized monoclonal IgG4 antibody targeting HERV-W-Env [30, 31]. For the first time in human therapeutics, this new drug is targeting a HERV protein, which could represent an upstream pathogenic driver leading to MS. Temelimab has already demonstrated an excellent safety profile and promising results in neuroprotection and in myelin preservation in a phase II clinical trial (NCT03239860) [26••, 30].

This example of HERV-W and MS illustrates how fundamental research can lead to new therapeutic strategies. In MS, approved drugs mainly consist of immunomodulatory agents, the efficacy of which has only been shown to alleviate relapses of early symptoms in most MS cases, but were shown to be ineffective at stalling disease progression. The involvement of HERV-W has open new therapeutic avenues addressing unmet needs, since acting on pathogenic mechanisms involved in lifelong disease progression, demyelination, and neurodegeneration [26••, 30].

#### HERV-K and Amyotrophic Lateral Sclerosis

ALS is another example of how a complex disease of unknown etiology can be analyzed from a new point of view thanks to the demonstration of HERV-K involvement in its pathogenesis, thereby unveiling a new pathogenic target for this rapidly fatal disease.

ALS is a neurodegenerative disorder primarily affecting motor neurons, leading to severe motor disabilities and eventually to death, mainly from ventilatory failure within 2 to 3 years following symptoms onset [32]. Two main forms of the disease are described, a familial form and a sporadic form. Genetic mutations are causative in familial forms accounting for about 10% of ALS patients. For the 90% of ALS patients affected by the sporadic form, the etiology of the disease remains elusive. Hypotheses have incriminated environmental factors including smoking and exposure to agricultural chemicals and heavy metals [33]. Infections from exogenous viruses have also been considered as a risk factor for developing ALS, such as herpesvirus and enterovirus infections [33]. However, none of these environmental factors have been definitively shown to be a causative factor, either because of the scarcity of the data or because of conflicting results [33].

ALS has recently been associated with HERV-K, and particularly with its envelope protein HERV-K-Env. HERV-K transcripts and proteins have been detected in human ALS brain tissue, as well as reverse transcriptase activity in blood [34, 35]. In vitro, HERV-K-Env induced neurotoxicity and its expression in human neurons caused inhibition of neurite growth and cell death [35]. Transgenic mice expressing the HERV-K*-*Env displayed volume loss in the motor cortex and decreased activity in pyramidal neurons, while they developed progressive motor dysfunction mimicking the clinical presentation of human ALS [35].

Concerning the activation of HERV-K expression in ALS, pro-inflammatory cytokines such as TNF-α have been shown to enhance HERV-K transcription and protein production in human astrocytes and neurons [36]. Mechanistically, the presence of functional ISREs (interferon-stimulated response elements) in HERV-K LTR allows the binding of NF-κB (nuclear factor kappa B) and IRF1 (interferon regulatory factor 1), further driving the activation of transcription [36, 37].

As presented in this section with best-studied examples to date, the involvement of HERV in diseases of formerly unknown etiology has brought new paradigms for tackling such disorders. The next part of this review will examine evidence that HERV is involved in the pathogenesis of type 1 diabetes, which could open up new therapeutic opportunities for treatment.

## The Elusive Etiology of T1D: an Interplay Between Environmental Viruses and HERV?

### T1D and Environmental Viruses

#### Detection of the Virus Per Se in T1D Patients

Several environmental viruses have already been associated with T1D, but most of the results were later challenged or are still awaiting confirmation. The most robust association between T1D and environmental viruses relates to enteroviruses, and especially to coxsackieviruses B (CV-B). Enterovirus potentially interact with several receptors [38], among which CAR (coxsackie adenovirus receptor) is the most studied in the T1D context. Enteroviruses are able to infect pancreatic islets via CAR, which is expressed on beta cells and alpha cells, and are able to replicate in both of these cell types [39, 40]. However, histology studies have mainly detected the VP1 (viral protein 1) capsid protein in pancreatic beta cells [41, 42]. The association between enteroviral infection and T1D has also been observed in many instances by molecular and immunological techniques in various biological samples, especially in the blood compartment and in the pancreas [42–44]. Thus, the presence of enteroviruses in T1D patients was observed by multiple groups, with different techniques and in several human tissues.

#### Detection of Viral Sensors and Antiviral Signature in T1D Patients

Upon virus entry or when exposed to viral molecules, cells will mount responses starting by the detection of the infection. PKR (protein kinase R) and MDA5 (melanoma differentiation–associated protein 5) are involved in viral RNA sensing. PKR has been detected in islets of T1D patients where it co-localizes with VP1 expression [41, 45], and single nucleotide polymorphisms within the gene encoding MDA5 have been found associated with higher risk of developing T1D [46]. The transcription factor STAT-1 (signal transducer and activator of transcription 1), which is involved in antiviral response, has also been found to be upregulated in insulin-containing islets of T1D [47]. Of note, STAT-1 expression is strongly correlated with hyperexpression of HLA Class I antigens, a characteristic feature of T1D pathogenesis [47]. Antiviral antibodies against EBV antigens have also been found to be associated with T1D in an immunoproteomic array profiling antiviral antibodies targeting 23 viral strains [48]. In this study, antiviral antibodies against 646 viral antigens were screened using a high-throughput immunoproteomics approach in patients with new-onset T1D. Among the viral strains tested were the six exogenous viruses epidemiologically associated with T1D (CMV, EBV, CV-B, rubella virus, mumps virus, and rotaviruses) and the endogenous retrovirus HERV-K [48].

Thus, the detection of recent or past viral infections, either directly or indirectly as described above, has provided converging evidences of particular infectious profiles in T1D patients [49••]. However, whether enteroviruses, or possibly other viruses like EBV, are inducers or aggravating factors of T1D is still an open question. The next paragraph will examine pathogenic mechanisms affecting pancreatic cells upon viral infection.

#### Involvement of Viral Infection in T1D Pathogenesis

An interplay between enteroviruses, innate and adaptive immunity may result in various mechanisms by which environmental viruses can contribute to T1D development [50••].

##### Direct Pancreatic Insults Following Enteroviral Infections

Human pancreatic islets, and beta cells in particular, can sustain a persistent CV-B infection [51]. Enteroviral infections were reported to induce beta cell death, decrease in insulin mRNA expression and insulin secretion and disruption of Golgi apparatus [52, 53]. In vitro, ductal cells can also be persistently infected with the diabetogenic CV-B4 strain (CV-B4 E2) thus impairing the differentiation of precursor endocrine cells and disturbing the microRNA expression profile [54, 55••].

##### Innate Immune Cells Entanglement in Deleterious Effects of Enteroviral Infections

Monocytes and macrophages are major target cells of enteroviruses, which upon infection induce expression of interferons and pro-inflammatory cytokines such as TNF alpha and IL-6 [56, 57, 58••]. Beta cells are particularly sensitive to interferons, which can induce ER stress, reduce insulin content, increase proinsulin to insulin ratio, and decrease expression of PC1 (proinsulin convertase 1) and PC2 (proinsulin convertase 2) enzymes responsible for maturation of proinsulin into insulin [59]. The pro-inflammatory state of beta cell resulting from viral infection is then amplified by the recruitment and activation of monocytes and dendritic cells, the secreted chemokines and cytokines of which will participate to the recruitment of T cells, including autoreactive clones [60, 61].

##### Peculiar Role of Facilitating Antibodies

Following the initial contact of a virus with a host, antibodies targeting the invader will be produced upon activation of the adaptive immune system. Unfortunately, some of these antibodies will not prevent a subsequent infection, but will rather increase it [62••]. This phenomenon is termed ADE (antibody-dependent enhancement) and relies on so called “facilitating antibodies.” Facilitating antibodies targeting the CV-B VP4 (viral protein 4) capsid protein have been detected in T1D patients and were reported to enhance the infection of monocytes and macrophages by CV-B4, which could promote dissemination of the virus to the pancreas [63–65]. The infection of monocytes and macrophages with CV-B4 in the presence of facilitating antibodies was shown to induce the production of IFN-α (interferon alpha), which can stimulate the presentation of autoantigens from local tissue lesions, and the secretion of pro-inflammatory cytokines [66–68]. The pathogenic effects of facilitating antibodies have also been observed in mice, in which anti-CV-B4 facilitating activity of the serum was observed upon the inoculation of CV-B4 [69]. Mice that were subsequently re-infected with this virus presented elevated blood glucose level and pancreas damage. This correlated with an increased viral load of the pancreas, compared with controls [70]. The ADE process enhancing, or even allowing, the infection of monocytes and other cell types may represent a critical step for a significant contribution of enterovirus to the pathogenesis of T1D [62••].

However, commonly described consequences of environmental virus infections, including the direct detrimental effect on beta cells and the potential indirect stimulation of autoimmunity, still do not provide a comprehensive understanding of the natural history of T1D with a prodromal period, a rather sudden clinical onset and a lifelong variable evolution. Thus, a causal evidence between environmental viruses and T1D remains elusive, seemingly indirect or requiring complementary effects from other pathogenic factors. Relevant to this matter, it is now well established that environmental factors such as viral infections may trigger the expression of HERV proteins. In the next sections, we will discuss how HERV may represent the missing link at the genetic interface between (i) environmental factors such as viruses and (ii) downstream activation of pathways resulting in T1D onset and progression.

### T1D and Endogenous Retroviruses

Genetic predisposition and numerous environmental factors, including viruses, appear to contribute to T1D pathogenesis [71]. In the absence of direct causal evidence between these contributing factors and beta cell destruction, HERV elements opened a new avenue of research with their unique positioning in the human genome and their interactions with environmental factors, particularly viruses. The current section will explain (i) how HERV could be transactivated in a T1D context, (ii) the animal evidences of ERV (endogenous retrovirus) involvement in disease pathogenic mechanisms, and finally (iii) the potential contribution of HERV in human T1D.

#### Transactivation of HERV by Exogenous Viruses in a T1D Context

Among the viruses that have been associated with T1D, even with low degree of confidence, only EBV has so far been described to transactivate HERV copies. As mentioned above, among 23 viral strains, only antiviral antibodies against EBV antigens have been found to be associated with T1D patients [48]. In parallel, EBV is described to transactivate both HERV-K18 and HERV-W [13, 14]. In particular, the EBVgp350 protein was shown to activate HERV-W in B cells and monocytes [14]. Moreover, EBV is able to demethylate DNA, thereby unlocking the epigenetic silencing of HERV [8]. Enteroviruses may also be involved in HERV transactivation, which is currently under investigation.

#### Animal Studies

Several studies reported an association between endogenous retroviruses and T1D pathogenesis in NOD mice. ERV copies, proteins, and particles have been observed in beta cells from NOD mice and have been suggested to be involved in this T1D mouse model [72–74]. Since these early reports, it has been shown that NOD mice developed autoantibodies against ERV envelopes proteins, with antibody titers increasing with age and disease progression [75••]. ERV proteins encoded by the *gag* gene (retroviral capsid proteins) also appeared to be involved in pro-inflammatory responses and in the induction of autoreactive T cells in NOD mice [75••].

Apart from the few evidences linking ERV to T1D in the NOD mouse model, two human endogenous retroviruses, HERV-K and HERV-W, have been presented as abnormally expressed in human T1D.

#### HERV-K

A low number of HERV-K copies within the C4 gene cluster of T1D patients’ genome has been linked to an increased risk of developing T1D. “Missing” protective endogenous retroviral insertions may thus suppress genetic factors of resistance against T1D [76]. Such protective endogenous retrovirus copies are well known in families of animal ERV and were shown to provide protection against infections by related retroviruses [77]. Antibodies directed against a peptide of HERV-K6 envelope protein have also been found elevated in a Polish cohort of young T1D patients [78]. HERV-K18 was claimed to be associated with T1D in human [79, 80], but this has later been disproved by successive studies [81–84]. Conrad and colleagues had described an endogenous retroviral sequence, termed IDDMK(1,2)22 and related to HERV-K18 family, encoding a protein with superantigen characteristics in T1D patients [80]. However, it turned out that the PCR-amplified sequences attributed to T1D probably came from genomic DNA contamination [81–83]. Still, an association between HERV-K18 polymorphisms and T1D was later reported [79] but was also subsequently rebutted [84].

#### HERV-W

The most compelling evidence for a functional association between HERV and T1D has been shown for HERV-W. The study of this endogenous retrovirus was initiated by the isolation of retroviral particles from MS patients [85]. Subsequent studies revealed that these particles originated from HERV elements, first termed multiple sclerosis associated retrovirus (MSRV). This prototype MSRV sequence later unveiled multiple copies of a previously unknown HERV family, now named HERV-W since using tryptophane (W) tRNA as a primer for reverse transcription [86].

The envelope protein of HERV-W, termed HERV-W-Env, has been detected in T1D patients and particularly in pancreatic acinar cells located in the vicinity of pancreatic lesions of T1D patients [87••]. Immunohistology of pancreata from nPOD repository suggested a spreading of HERV-W-Env expression paralleling disease progression and/or duration [87••], as already shown in MS post-mortem brains [25]. Antibodies directed against a peptide of HERV-W-Env have also been detected in serum of youths at risk for T1D and youths affected by T1D [78]. It appeared that elevated levels of these anti-HERV-W-Env antibodies overlapped with or preceded the appearance of conventional T1D autoantibodies (ICA, IAA, IA2A, and/or GADA) [78]. It has been observed that HERV-W-Env promoted macrophages recruitment within the pancreas and beta cell dysfunction, as shown by the inhibition of insulin secretion by HERV-W-Env in primary cultures of human islets of Langerhans [87••]. Of note, beta cells express TLR4 [88, 89] and HERV-W-Env exerts its pathogenicity through interaction with TLR4 [27].

Activation of TLR4 by chronically expressed HERV-W-Env is particularly intriguing in a T1D perspective. The stimulation of TLR4 has already been described to inhibit insulin secretion, to decrease beta cell viability and expression of PDX-1 and Maf-A, two critical transcription factors required for beta cell function [88, 89]. Additionally, downstream elements of TLR4 signaling are upregulated in T1D patients, such as NF-κB, MyD88, and TRIF [90].

Apart from its deleterious effects on beta cells, HERV-W-Env displays other pathogenic properties that may be relevant in T1D pathogenesis. In particular, HERV-W-Env impairs TLR4-expressing cells such as endothelial cells [91] and Schwann cells [92], both cell types whose dysfunction is associated with major T1D comorbidities. Moreover, a functional implication of HERV-W in immune processes has been evidenced. Pro-inflammatory cytokines expression was induced in monocytes upon stimulation with HERV-W-Env through TLR4 receptor activation, while dendritic cells were elicited to support a Th1-like type of T-helper lymphocyte differentiation [28••, 93, 94]. Likewise, HERV-W-Env has been shown to induce T cell responses with superantigen characteristics [95], which would provide an explanation for in vivo promotion of autoimmunity [28••].

The particular HERV-W-*env* genomic copy encoding the pathogenic HERV-W-Env protein involved in T1D remains to be identified. This identification is precluded by extensive interindividual variations existing in the proviral content of some HERV families in human genomes [96]. Such non-ubiquitous copies or somatic DNA rearrangements in chromosomal HERV sequences may explain difficulties to pinpoint the relevant copy in DNA databases of reference human genomes [96]. The identification of a peculiar HERV copy clustering with T1D, or T1D subtypes, would allow the identification of a potentially critical genetic risk factor that could not have been pinpointed from traditional genetic studies in which this part of the human DNA is not examined with appropriate methods required for multicopy transposable elements [4, 96••].

From the data presented in this review, it appeared that HERV-W-*env* could contribute to the genetic susceptibility to T1D and MS as encoding a key player at the gene-environment interface of their respective pathogenesis. Whether the genomic copies involved in the two diseases are identical or different has to be determined, but most of epidemiological studies about T1D and MS co-occurrence found higher prevalence rates than expected for one disease alone [97]. This observation contradicts studies on HLA risk haplotypes for MS that are protective from T1D [97]. This would be compatible with a dominant effect of the HERV-W factor over the HLA phenotype in a multiparametric equation predicting disease susceptibility. Within this equation, if HERV-W-*env* genomic copies involved in T1D and MS are identical, then the question of the disease onset in an organ instead of the other is raised. The virus tropism for the intestine and the pancreas (Enteroviruses) or for the central nervous system (Herpesviruses) could determine a local autoimmune reaction and a local direct toxicity occurring in the pancreas or in the brain, via HERV-W-Env transactivation.

**Fig. 3:**
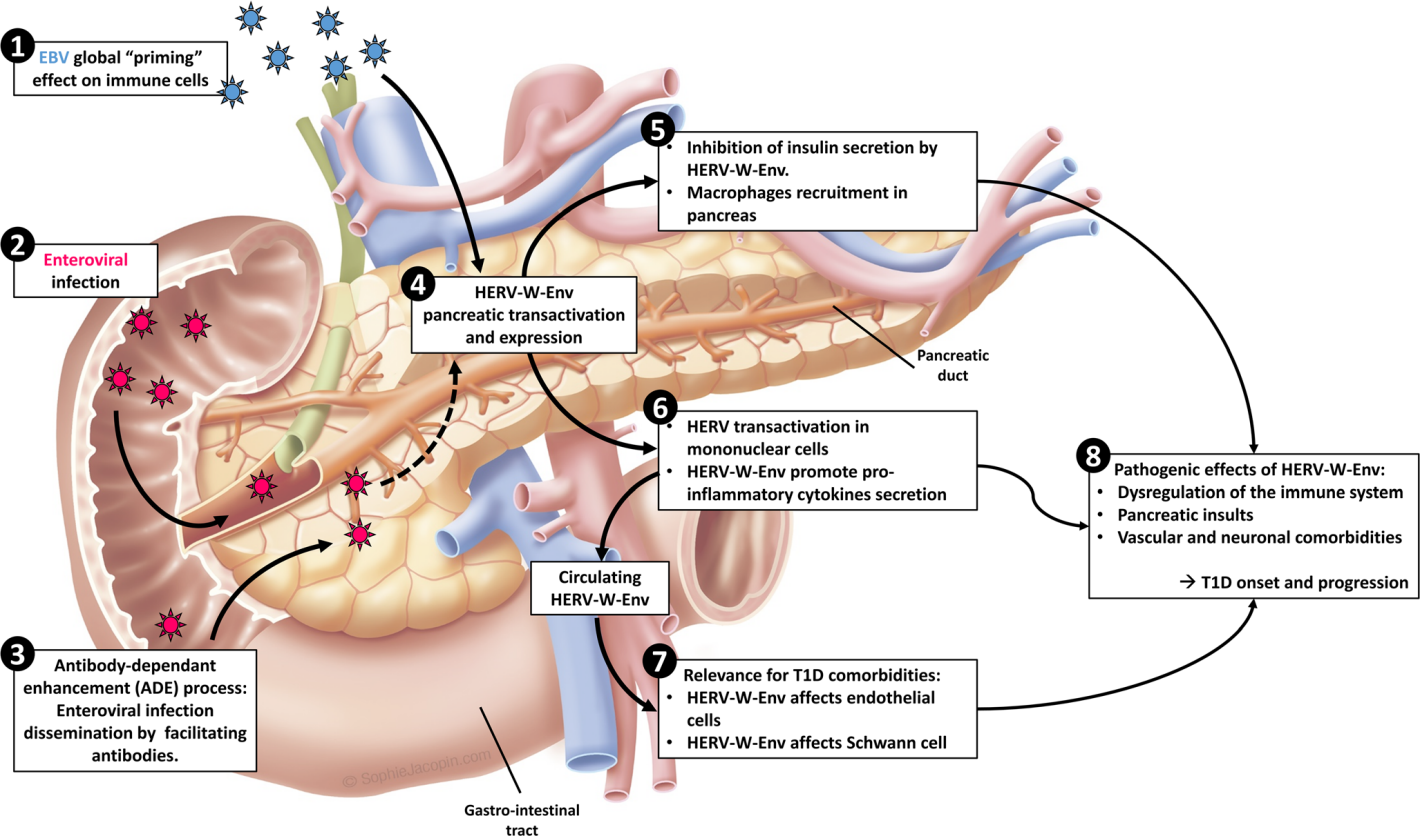
Global model depicting HERV-W-Env involvement in T1D onset and progression. Central to this model is the detection of HERV-W-Env, either locally in pancreatic acinar cells, or in systemic circulation (expressed by PBMC or present in a soluble form in blood) [87••]. Whether the soluble form of HERV-W-Env arises from pancreas or immune cells remains to be determined. ❶ In this model, EBV infection could be an early global priming event of immune cells. Accordingly, anti-EBV antibodies have been detected in T1D patients [48], and EBV can dysregulate epigenetic control [8], thereby rendering genomic region bearing HERV copies accessible to transcription [14]. ❷ Later in individual’s life, an enteroviral infection may activate directly HERV-W in the pancreas or in immune cells previously primed and harboring newly accessible DNA regions. The transactivation of HERV-W-Env by enteroviruses is a hypothesis currently under investigation. ❸ Involvement of enteroviral infection may be reinforced by the ADE (antibody-dependent enhancement) process and the production of facilitating antibodies, which increase enteroviral infection in monocytes and macrophages, resulting in infection spreading to the pancreas [50••]. ❹ HERV-W-Env transactivation and expression could occur in pancreatic acinar cells, ❺ resulting in inhibition of insulin secretion and macrophages recruitment within the pancreas [87••]. ❻ HERV-W-Env promotes pro-inflammatory cytokine production by immune cells [27] and its expression could occur in mononuclear cells thereby participating in increased circulating levels of HERV-W-Env in blood [14]. ❼ HERV-W-Env also exerts detrimental effects on endothelial cells [91] and Schwan cells [92], suggesting a possible implication in T1D-associated comorbidities. ❽ Altogether, these pathogenic effects argue for an involvement of HERV-W-Env in T1D onset and progression

Altogether, these data provide substantial arguments to consider HERV-W-Env at the interface between genetic susceptibility, environmental factors, and downstream pathogenic cascades leading to T1D. Figure 3 presents a scenario for the potential contributions of exogenous viruses and HERV into T1D pathophysiology. Briefly, particular HERV-W-*env* genomic copies could be activated by environmental infections, potentially recurrent and requiring an ADE facilitating process. Once activated and produced, HERV-W-Env protein would affect several targets, such as pancreatic beta cells, immune cells, and other TLR4-positive cells. This unique positioning identifies HERV-W-Env as a new therapeutic target in T1D. A phase IIa clinical trial addressing HERV-W-env-neutralizing humanized antibody temelimab clinical safety and pharmacokinetics endpoints in T1D adults patients has recently been conducted (ClinicalTrials.gov Identifier: NCT03179423) [98]. It has confirmed the very strong safety profile of temelimab and now calls for further clinical developments in recent-onset T1D pediatric patients.

## Conclusion

The present review discussed the largely disregarded aspects of HERV multicopy elements which may be dysregulated by epigenetic changes and transactivated by environmental triggers, possibly resulting in autoimmune diseases such as T1D and MS, or degenerative diseases such as ALS.

In this scenario, successive events would link external stimuli, like enteroviruses in the case of T1D, to pathological cascades associated with HERV elements in susceptible tissues. This would provide possible solutions to the multifactorial equation of these complex diseases and this has already prompted different groups to evaluate novel therapeutic approaches in patients with MS or T1D, in which HERV-W is involved [99] or in patients with ALS in which HERV-K is involved [100].

This area of research is rapidly evolving and it is likely that HERVs, representing about 8% of the human genome, may contribute to T1D and other complex and multifactorial human diseases. The therapeutic approaches targeting toxic proteins expressed by HERVs may thus offer completely novel perspectives of treating upstream pathogenic factors without affecting downstream dysregulated physiological functions.
